# Exosomal circ_0026611 contributes to lymphangiogenesis by reducing PROX1 acetylation and ubiquitination in human lymphatic endothelial cells (HLECs)

**DOI:** 10.1186/s11658-022-00410-z

**Published:** 2023-02-17

**Authors:** Wenjian Yao, Xiangbo Jia, Li Zhu, Lei Xu, Quan Zhang, Tian Xia, Li Wei

**Affiliations:** 1 Department of Thoracic Surgery, Henan Provincial People’s Hospital, People’s Hospital of Zhengzhou University, School of Clinical Medicine, Henan University, No.7, Weiwu Road, Jinshui District, Zhengzhou, 450003 Henan China; 2 Department of Thoracic Surgery, Zhengzhou University People’s Hospital, Henan Provincial People’s Hospital, Zhengzhou, 450003 Henan China

**Keywords:** Esophageal squamous cell carcinoma, Exosomes, circ_0026611, PROX1, Lymphangiogenesis

## Abstract

**Background:**

Esophageal squamous carcinoma (ESCC) is a common malignancy that originates in the digestive tract. Lymph node metastasis (LNM) is a complicated process, and tumor lymphangiogenesis has been reported to be associated with the spread of tumor cells to lymph nodes (LNs), including in ESCC. However, little is currently known about the mechanisms involved in lymphangiogenesis in ESCC tumors. According to previous literature, we know that hsa_circ_0026611 expresses at a high level in serum exosomes of patients with ESCC and shows a close association with LNM and poor prognosis. However, details on the functions of circ_0026611 in ESCC remain unclear. We aim to explore the effects of circ_0026611 in ESCC cell-derived exosomes on lymphangiogenesis and its potential molecular mechanism.

**Methods:**

We firstly examined how circ_0026611 may express in ESCC cells and exosomes by quantitative reverse transcription real-time polymerase chain reaction (RT-qPCR). The potential effects circ_0026611 may exert on lymphangiogenesis in ESCC cell-derived exosomes were assessed afterward via mechanism experiments.

**Results:**

circ_0026611 high expression pattern was confirmed in ESCC cells and exosomes. ESCC cell-derived exosomes promoted lymphangiogenesis by transferring circ_0026611. Besides, circ_0026611 interacted with *N*-α-acetyltransferase 10 (NAA10) to inhibit NAA10-mediated prospero homeobox 1 (PROX1) acetylation with subsequent ubiquitination and degradation. Furthermore, circ_0026611 was verified to promote lymphangiogenesis in a PROX1-mediated manner.

**Conclusions:**

Exosomal circ_0026611 inhibited PROX1 acetylation and ubiquitination to promote lymphangiogenesis in ESCC.

**Graphical Abstract:**

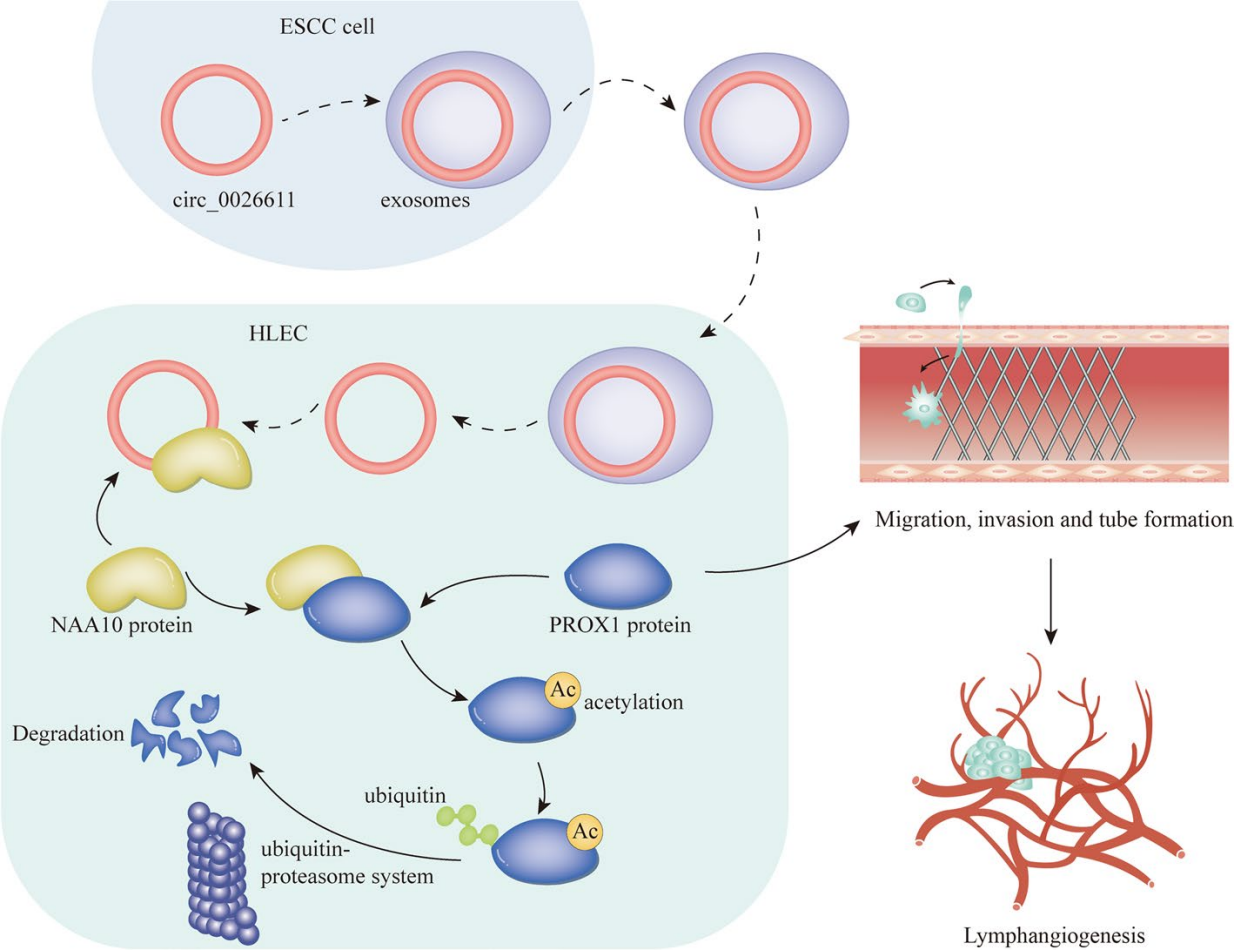

**Supplementary Information:**

The online version contains supplementary material available at 10.1186/s11658-022-00410-z.

## Background

Esophageal squamous carcinoma (ESCC) represents a major type of esophageal cancer that arises from epithelial cells of the esophagus and is characterized by late-stage diagnosis and frequent recurrence [1]. It has been reported that over half of newly diagnosed ESCC cases occur in China [2]. Despite advances such as chemotherapy and radiotherapy made in the treatment of ESCC, the overall management of this human malignancy remains challenging due to limited effective therapeutics [3]. Local lymph node metastasis (LNM) is a typical sign of failure for ESCC clinical treatments, and it has been reported that more than half of patients with ESCC have a locally advanced status with LNM when the malignancy is diagnosed, which results in poor prognosis [4]. Moreover, prediction of LNM in ESCC has been demonstrated to have prognostic significance [5, 6]. Therefore, elucidating LNM-related genomic alterations and identifying the underlying molecular mechanisms may provide better clinical strategies for ESCC treatment.

LNM represents a complicated process regulated by multiple factors, including intratumoral and peritumoral lymphangiogenesis, tumor cell proliferation to lymphatic vessels, tumor cell transport to lymph nodes (LNs) via the lymphatic system, and tumor cell expansion in LNs [7]. Among these factors, lymphangiogenesis can be considered as the formation of new lymphatic vessels on the existing lymphatic network. Lymphatic endothelial cells go through proliferation, germination, and migration to form new tumor lymphatic vessels [8]. It has been reported that tumor lymphangiogenesis is associated with lymphatic metastasis in cancer [9, 10], including in ESCC [11]. However, at present, little is known about the mechanisms involved in lymphangiogenesis in ESCC tumors.

Circular RNAs (circRNAs) are a class of noncoding RNA with well-known significance in modulating a broad range of cancers [12], including ESCC, and many circRNAs have gradually been identified as potential biomarkers for treating ESCC, such as circGSK3β [13], hsa_circ_0006948 [14], and circNTRK2 [15]. A recent report illustrates that serum exosomal has_circ_0026611 was significantly upregulated in ESCC with LNM and also indicated poor prognosis [16]. On the basis of this information, we intended to determine whether this circRNA may have certain impacts on the progression of ESCC.

Exosomes are a type of microvesicle (MV) with a typical diameter of 30–100 nm. They can deliver proteins, lipids, lncRNAs, circRNAs, and microRNAs, thereby promoting effective intercellular communication [17, 18]. Tumor-derived exosomal hsa-circ-0048117 has been reported to facilitate M2 macrophage polarization in ESCC [19]. Therefore, we also tried to verify the effect of exosomal circ_0026611 in the progression of ESCC.

In summary, this study aims to explore the effects of circ_0026611 in ESCC cell-derived exosomes on lymphangiogenesis and its potential molecular mechanism.

## Methods

### Cell culture

ESCC cell lines KYSE30 (BW-S1092), KYSE150 (HT-X2475), EC109 (BW-5999), and EC9706 (BW-6316) were obtained from Cell Bank of Chinese Academy of Sciences (Shanghai, China) and kept in RPMI-1640 medium (#11875-093, Gibco, Grand Island, NY, USA) containing 10% fetal bovine serum (FBS; #16000-044, Grand Island, NY, USA) at 37 °C in a 5% CO_2_ cell culture incubator. Human lymphatic endothelial cells (HLECs, CP-H026, Procell) were obtained from ScienCell Research Laboratories (Carlsbad, CA, USA) and cultured in endothelial cell medium (ECM) with 5% FBS and endothelial cell growth medium supplements (CC-3121, Lonza, Basel, Switzerland). Immortalized esophageal epithelial NE-1 cell line (CP-H031, Procell) was obtained from State Key Laboratory of Oncology in South China, Sun Yat-sen University Cancer Center and cultured in Dulbecco’s modified Eagle medium (DMEM; #11885-076, Gibco, Grand Island, NY, USA) with 10% FBS in a 5% CO_2_ atmosphere at 37 °C.

### Cell transfection

Specific short hairpin RNAs (shRNAs) targeting circ_0026611 (sh-circ_0026611#1/2) and PROX1 (sh-PROX1#1/2) together with their negative control shRNA (sh-NC) were constructed to silence circ_0026611 or PROX1 expression. For the overexpression of circ_0026611 and PROX1, the whole sequences were synthesized and subcloned into pcDNA3.1 vector, with pcDNA3.1 empty vector as the negative control (vector). In line with the supplier’s protocols, transfections were conducted with Lipofectamine 2000 (Invitrogen). Related sequence information is included in Additional file 2: Table S1.

### Quantitative reverse transcription real-time polymerase chain reaction (RT-qPCR)

Total RNA of cells was extracted by using TRIzol reagent (#15596018, Invitrogen, Carlsbad, CA, USA). Synthesis of complementary DNA (cDNA) for circRNAs and messenger RNAs (mRNAs) was synthesized by SuperScript III First-Strand Synthesis SuperMix (11752050, Invitrogen, Carlsbad, CA, USA). RT-qPCR reaction was achieved with SYBR Green Kit (#1725085, Bio-Rad Laboratories, Hercules, CA, USA) and real-time PCR system (ABI 7500, Thermo Fisher, Rockford, IL, USA) followed by 2^−ΔΔCt^ method. Glyceraldehyde-3-phosphate dehydrogenase (GAPDH) and U6 were considered as internal controls in this process.

### Exosome isolation

Exosomes were isolated from supernatants of NE-1, KYSE30, and EC109 cells using ultracentrifugation methods. In brief, the cells were cultured in a complementary medium until 80% confluence, and then the medium was replaced with the defined medium without FBS. After 2 days of culture, the supernatants were harvested and centrifuged at 300*g* for 15 min, 2000*g* for 15 min, and 10,000*g* for 30 min. The supernatants were filtrated through a 0.22 μm polyvinylidene fluoride (PVDF) filter (Millipore, USA). Then the supernatants were collected to isolate exosomes by ultracentrifugation at 120,000*g* for 70 min (Beckman Coulter) twice. Particle Metrix (PMX), transmission electron microscopy (TEM), and western blotting were used to identify the exosomes.

### Tube formation assay

Serum-free ECM starved human umbilical endothelial cells (HUVECs) were planted into 24-well plates coated with Matrigel (BD Biosciences) at a density of 2 × 10^4^ cells per well. Conditional medium was collected by incubating exosome-treated or transfected HLECs cells with serum-free Dulbecco’s modified Eagle medium (DMEM) for 24 h and added into each well at 200 μl per well. The images of tube structure were captured under a light microscope, and tube formation was quantified with ImageView 3.7 (Jingtong, China).

### Western blot

Total protein extracted from ESCC cell lines (KYSE30-Exos and EC109-Exos) was isolated by radio-immunoprecipitation assay (RIPA) buffer, and after being separated through sodium dodecyl sulfate–polyacrylamide gel electrophoresis (SDS–PAGE), proteins were transferred to PVDF membranes and cultured in 5% skim milk. The membranes were cultivated with primary antibodies overnight at 4 °C, followed by cultivation with secondary antibody for 1 h. After washing in Tris-buffered saline + Tween 20 (TBST), the secondary antibodies were added and finally assayed by enhanced chemiluminescence (ECL) substrate. The primary antibodies were as follows: anti-CD9 (ab92726, Abcam, Cambridge, MA, USA), anti-CD63 (ab134045), anti-β-tubulin (sc-166729, Santa Cruz Biotechnology, Santa Cruz, CA, USA), anti-VEGF-C (vascular endothelial growth factor C, #2445, Cell Signaling Technology, Boston, MA, USA), anti-VEGF-D (vascular endothelial growth factor D, sc-373866, Santa Cruz Biotechnology, Santa Cruz, CA, USA), anti-VEGFR3 (vascular endothelial growth factor receptor 3, sc-28297, Santa Cruz Biotechnology, Santa Cruz, CA, USA), anti-LYVE-1 (lymphatic vessel endothelial hyaluronan receptor 1, ab219556, Abcam, Cambridge, MA, USA), anti-podoplanin (#9047, Cell Signaling Technology, Boston, MA, USA), anti-PROX1 (prospero homeobox 1, sc-81983, Santa Cruz Biotechnology, Santa Cruz, CA, USA), and anti-GAPDH (ab9485, Abcam, Cambridge, MA, USA).

### Transwell assays

ESCC cells (KYSE30-Exos and EC109-Exos) were planted on the top of 24-well transwell chambers (#353097, BD Falcon, Bedford, MA, USA) coated with Matrigel (FAL354483, Corning, Shanghai, China) for invasion assay or without Matrigel for migration assay. The lower chambers were loaded with complete medium. Twenty-four hours later, cells in the upper layer were removed with caution by a cotton swab and then fixed in methanol solution for 15 min. Crystal violet was adopted to stain the membranes for 10 min, and the invaded or migrated cells were observed and counted under a microscope (10 × 10).

### Dual-luciferase reporter assay

VEGFR3 promoter was subcloned into the pGL3 luciferase vector (E1751, Promega, Madison, WI, USA) and then cotransfected with circ_0026611 and Vector into HLECs using Lipofectamine 2000. All luciferase intensities were examined by Dual Luciferase Report Assay System (E1910, Promega, Madison, WI, USA) 36 h after transfection.

### RNA pulldown assay

Biotinylated circ_0026611 was taken and treated with structure buffer to form a secondary structure. Streptavidin beads were added to biotin-labeled and denatured circ_0026611 and spun at 4 °C for 2 h. Cells were lysed with lysis buffer, and then the lysates were incubated with specific biotin-labeled probes for 2 h. After that, the mixtures were incubated with streptavidin-coated magnetic beads to pull down the biotin-labeled RNA complex. After washing, the RNA complex was extracted with TRIzol and then detected by western blot analysis. The experiments went through three independent repeats.

### RNA-binding protein immunoprecipitation (RIP) assay

With the EZMagna RIP kit (17-701, Sigma-Aldrich, St. Louis, MO, USA), RIP assay in HLECs was carried out with the specific antibodies and normal control anti-IgG antibody (CS200621, Millipore, Billerica, MA, USA). Lysates were obtained from HLECs using RIP lysis buffer. The lysis was incubated with the magnetic beads conjugated with the NAA10 antibody (#9046, Cell Signaling Technology, Boston, MA, USA) or IgG antibody (negative control). The precipitated RNAs were analyzed by RT-qPCR.

### Statistical analysis

All data from experiments including three biological replications are presented as mean ± standard deviation (SD). Data analysis was achieved by Student’s *t*-test and one-way/two-way analysis of variance (ANOVA), applying SPSS 19.0 software. **P < 0.01 indicates that related data contained statistical significance.

## Results

### circ_0026611 expressed highly in ESCC cells and its exosomes

**Fig. 1:**
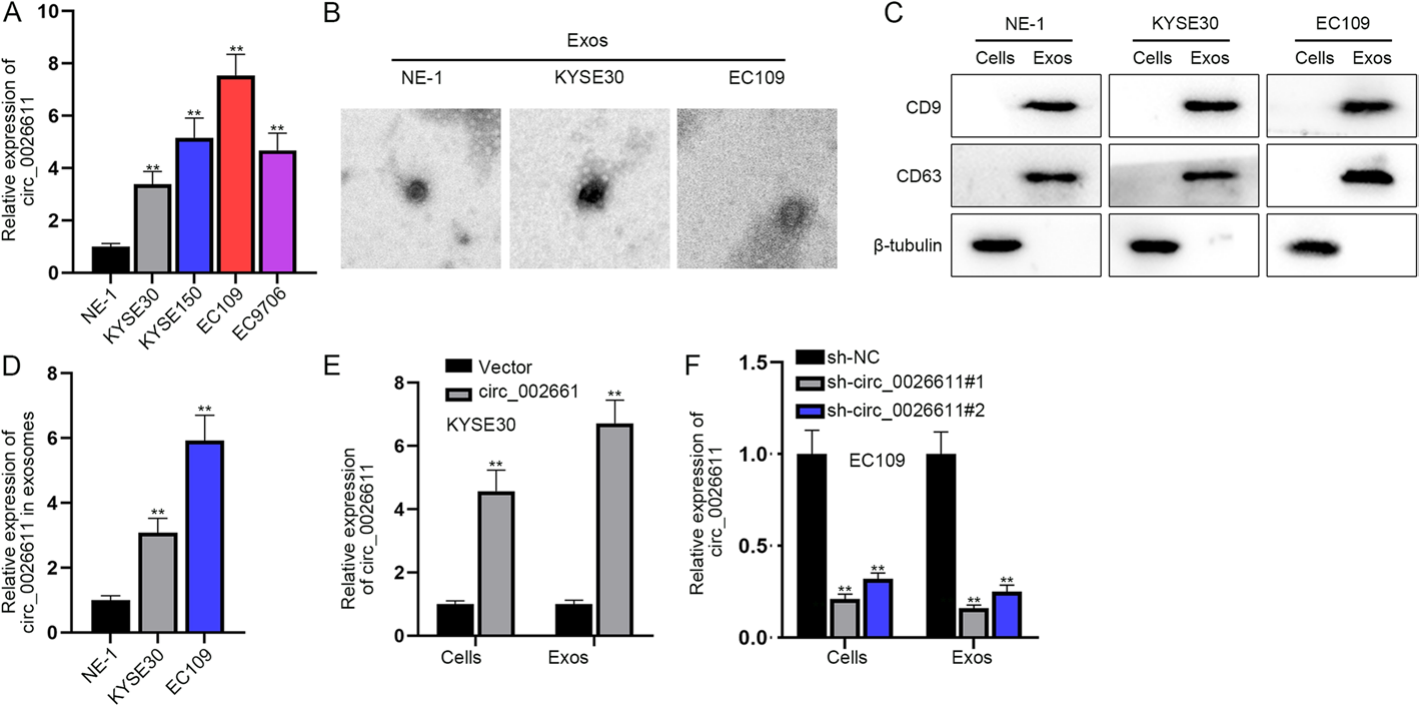
circ_0026611 expressed highly in ESCC cells and its exosomes. **A** RT-qPCR data of circ_0026611 expression in ESCC cells. **B** The exosomes of KYSE30 and EC109 cells were detected under electron microscope. **C** Protein expression of exosome-related markers in KYSE30/EC109 cells. **D** RT-qPCR data of circ_0026611 expression in ESCC-derived exosomes. **E** Expression of circ_0026611 in KYSE30 cells and KYSE30-derived exosomes was measured by RT-qPCR. **F** Expression change of circ_00266111 in EC109 cells and EC109-derived exosomes. **P < 0.01

Expression of circ_0026611 in ESCC cells was detected by RT-qPCR. We observed high circ_0026611 expression in the four ESCC cell lines (KYSE30, KYSE150, EC109, EC9706) compared with the normal control NE-1 cell line, with the highest expression in EC109 and the lowest in KYSE30 (Fig. 1A). Thus, KYSE30 and EC109 cell lines were chosen for further study. Then we observed the KYSE30-derived and EC109-derived exosomes under an electron microscope (Fig. 1B). It was found through western blot data that the extracted exosomes could express CD9 and CD63, indicating that the extracts were indeed exosomes (Fig. 1C). Next, we uncovered that the expression of circ_0026611 was higher in exosomes derived from KYSE30 or EC109 cell lines than from NE-1 cell line (Fig. 1D). We overexpressed circ_0026611 in KYSE30 cells and interfered with circ_0026611 in EC109 cells for further investigations, and RT-qPCR data rendered satisfactory efficiency under different transfection groups (Fig. 1E, F). In conclusion, circ_0026611 was upregulated in ESCC cells and cell-derived exosomes.

### ESCC cell-derived exosomes promoted the migration, invasion, and lymphangiogenesis of HLECs

**Fig. 2:**
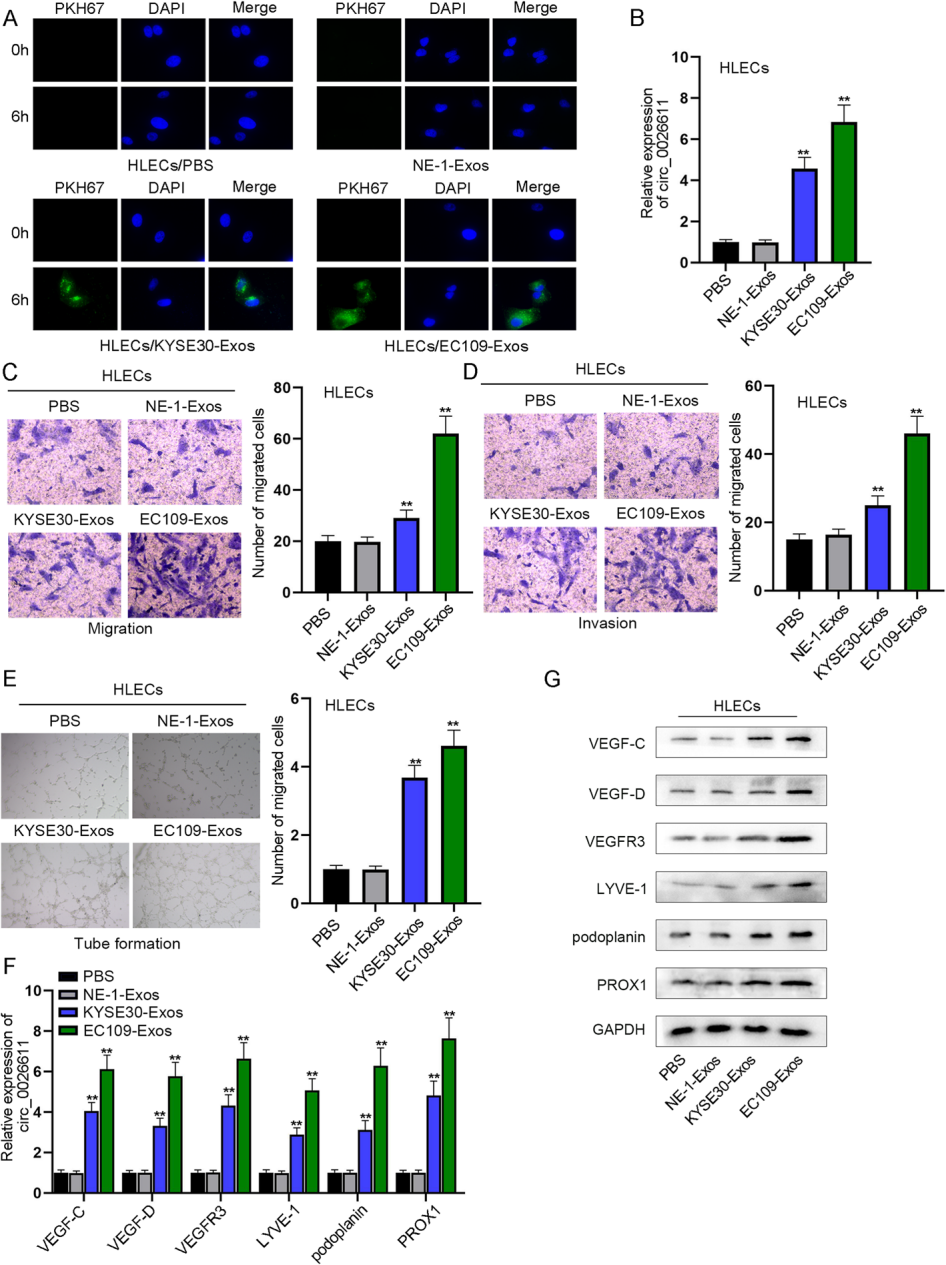
ESCC cell-derived exosomes promoted the migration, invasion, and lymphangiogenesis of HLECs. **A** Exosome labeling and tracking assay detected the absorption of exosomes from ESCC in HLECs. **B** circ_0026611 expression change after HELCs absorbing ESCC-derived exosomes was tested. **C**, **D** Transwell assays were conducted to determine the influence of treating ESCC-derived exosomes on HLEC migration and invasion. **E** Whether ESCC-derived exosomes may affect HLEC tube formation was verified by tube formation assay. **F**, **G** Expression of the lymphangiogenesis-related markers in HLECs was tested in different groups. **P < 0.01

Next explored the effect of ESCC cell-derived exosomes on lymphangiogenesis. Exosome labeling and tracking assay detected the absorption of exosomes from ESCC in HLECs. After 6 h of exosome treatment, PKH67-labeled exosomes from ESCC cells were absorbed into HLECs (Fig. 2A). In HLECs, circ_0026611 displayed significantly increased expression after the absorption of exosomes from KYSE30, and the upregulation was even more significant after the absorption of exosomes from EC109 (Fig. 2B). KYSE30-Exos-treated HLECs were found to promote the migration and invasion of the cells, and the promotion was more obvious under EC109-Exos treatment (Fig. 2C, D). Tube formation assay revealed that KYSE30-Exos promoted the tube formation of HLECs, while EC109-Exos promoted the formation more strongly (Fig. 2E). On the basis of the literature [20], we tested the influence of ESCC-derived exosomes on the expression of lymphangiogenesis-related markers (VEGF-C, VEGF-D, VEGFR3, LYVE-1, podoplanin, PROX1) in HLECs at the same time, which showed that they were increased under KYSE30-Exos treatment, and the increase was more significant under EC109-Exos treatment (Fig. 2F, G). To conclude, ESCC-derived exosomes promoted lymphangiogenesis.

### ESCC cell-derived exosomes promoted HLEC migration, invasion, and lymphangiogenesis by delivering circ_0026611

**Fig. 3:**
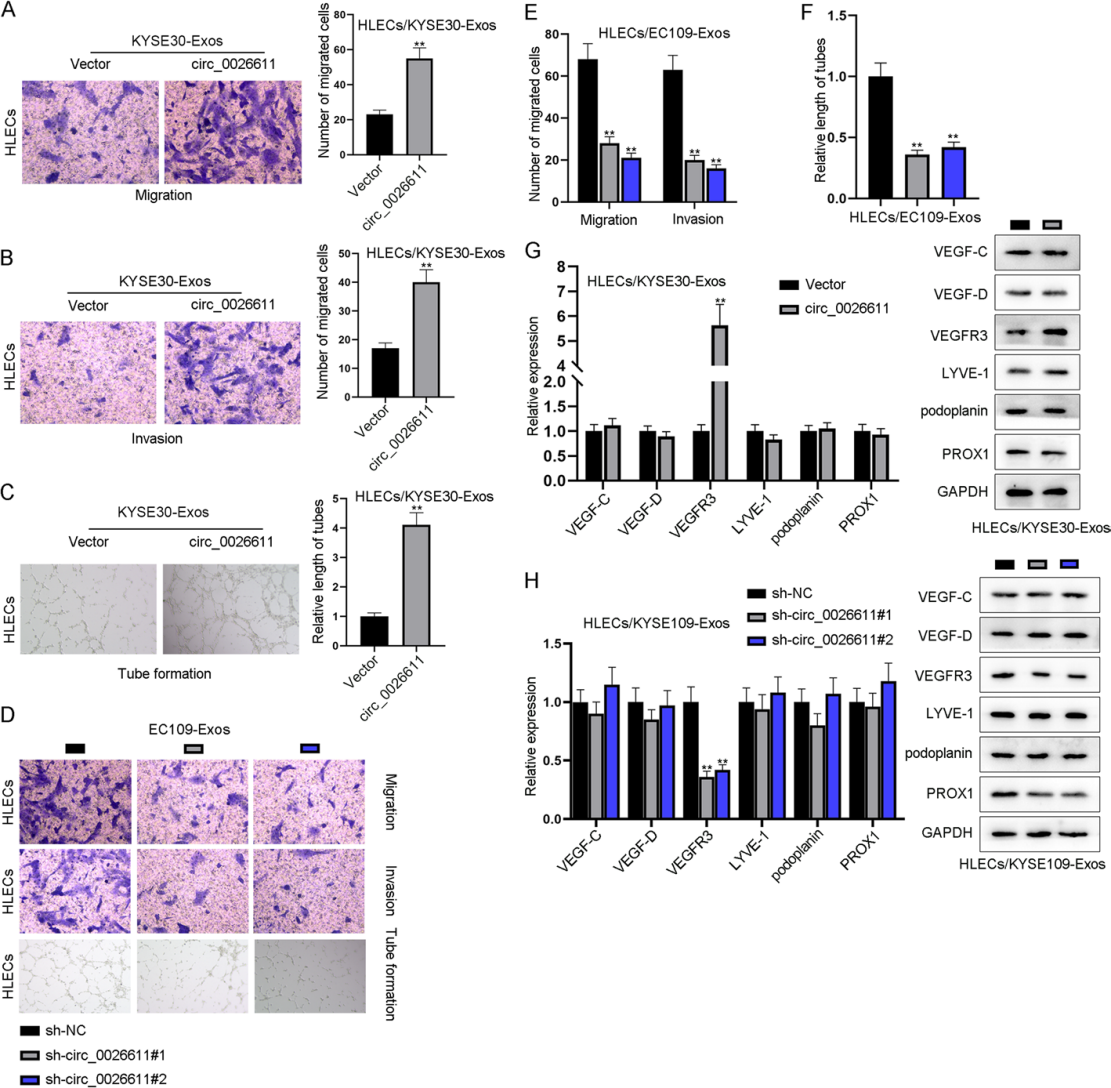
ESCC cell-derived exosomes promoted HLEC migration, invasion, and lymphangiogenesis by delivering circ_0026611. **A**–**F** Functional assays were carried out to evaluate the impact of circ_0026611-overexpressing ESCC-derived exosomes on the migration, invasion, and tube formation of HLECs. **G**, **H** The expression of lymphangiogenesis-related markers in different groups. **P < 0.01

To verify whether ESCC-derived exosomes may regulate HLECs via circ_0026611, we conducted Transwell assay as well as tube formation assay and tried to measure whether the overexpression or knockdown of circ_0026611 may affect the migration, invasion and tube formation of HLECs. We overexpressed circ_0026611 in KYSE30-derived exosomes and silenced its expression in EC109-derived exosomes. Results showed that the overexpression treatment of circ_0026611 expression could aggravate the migration, invasion as well as tube formation ability of HLECs, while the silenced circ_0026611 expression exerted opposite results (Fig. 3A–F). Finally, we confirmed with western blot that VEGFR3 mRNA was positively regulated by circ_0026611 (Fig. 3G, H). To sum up, ESCC-derived exosomes transmitted circ_0026611 to promote lymphangiogenesis in vitro.

### circ_0026611 indirectly upregulated VEGFR3 by regulating PROX1 protein after translation

We learned from the literature that VEGFR3 and PROX1 have an important impact on the process of lymphangiogenesis [21, 22]. It was also reported that PROX1 could regulate the transcription of VEGFR3 [23, 24]. Therefore, with the results above, we suggested that circ_0026611 might promote VEGFR3 expression through regulating PROX1 protein, thereby affecting lymphangiogenesis. At first, we observed through luciferase reporter assay that VEGFR3 promoter exhibited increased luciferase activity after the overexpression of circ_0026611, indicating that circ_0026611 promoted VEGFR3 transcription (Additional file 1: Fig. S1A). When PROX1 was interfered, the mRNA and protein expression of VEGFR3 decreased in HLECs (Additional file 1: Fig. S1B). Luciferase reporter assay was utilized to detect the influence of PROX1 to VEGFR3 transcription. The findings proved that PROX1 silencing inhibited VEGFR3 transcription (Additional file 1: Fig. S1C), and the intervention of PROX1 could completely inhibit the enhanced luciferase activity of VEGFR3 promoter induced by circ_0026611 overexpression (Additional file 1: Fig. S1D). Furthermore, PROX1 silencing could perfectly reverse the upregulated VEGFR3 expression caused by circ_0026611 overexpression (Additional file 1: Fig. S1E). Thus, we concluded that circ_0026611 affected VEGFR3 through PROX1. We continued to explore how circ_0026611 affected PROX1. From the results above (Fig. 3G, H), we found that circ_0026611 did not affect the expression of PROX1 mRNA but only positively regulated the expression of PROX1 protein, suggesting that circ_0026611 may influence PROX1 at the translation and post-translation levels. To determine at what level circ_0026611 affected PROX1 protein, we then performed CHX (protein synthesis inhibitor) and MG132 (proteasome inhibitor) assays. Western blot data showed that, when circ_0026611 was overexpressed, the degradation rate of CHX-treated PROX1 protein was decreased, while under the treatment of MG132, the overexpression of circ_0026611 seemed not to affect PROX1 protein level (Additional file 1: Fig. S1F,G). These results all indicated that circ_0026611 did not influence PROX1 protein synthesis, but it did inhibit the proteasomal degradation of PROX1 protein, thus positively regulating PROX1 protein. All in all, circ_0026611 indirectly promoted VEGFR3 expression by positively regulating PROX1 protein.

### ESCC exosomal delivered circ_0026611 promoted lymphangiogenesis by regulating PROX1

**Fig. 4:**
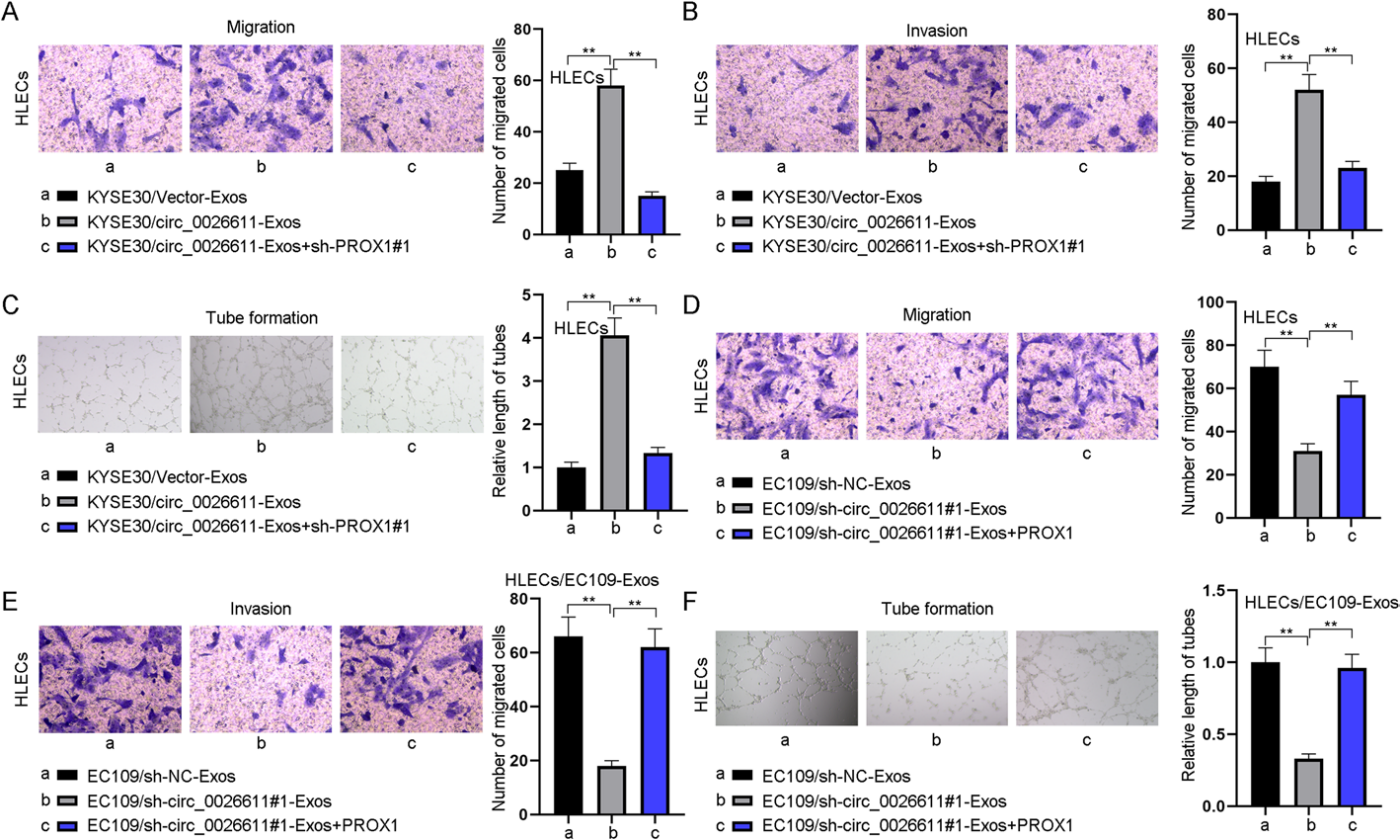
ESCC exosomal delivery circ_0026611 promoted lymphangiogenesis by regulating PROX1. **A**–**C** HLECs were divided into three groups. The first group was treated with exosomes derived from KYSE30 cells transfected with vector. The second and third groups were treated with KYSE30-derived exosomes with circ_0026611 overexpression vector, but the third group was also transfected with sh-PROX1#1 at the same time. The cell migration, invasion, and tube formation in the three groups were assessed via functional assays. **D**–**F** HLECs were divided into three groups. The first group was treated with exosomes derived from EC109 cells transfected with sh-NC. The second and third groups were treated with EC109-derived exosomes with circ_0026611 inhibiting vector, but the third group was also transfected with pcDNA3.1-PROX1 at the same time. Transwell assay and tube formation assay were carried out to detect the cell migration, invasion, and tube formation in the three groups. **P < 0.01

To verify whether circ_0026611 affected lymphangiogenesis through PROX1, we performed the following rescue assays. As shown in Fig. 4A–C, circ_0026611 overexpressed KYSE30-Exos promoted HLECs cell migration, invasion, and tube formation, but such effects were countervailed by silencing PROX1. Meanwhile, in rescue groups treated with silenced circ_0026611 expression, it was revealed that the capabilities to migrate, to invade, and to form tubes were inhibited in the circ_0026611-overexpressed KYSE30-Exos group, but the sh-PROX1#1 transfection could later compensate this impact to a normal level (Fig. 4D–F).

### NAA10 interacting with circ_0026611 mediated PROX1 acetylation and promoted its ubiquitination and subsequent degradation

**Fig. 5:**
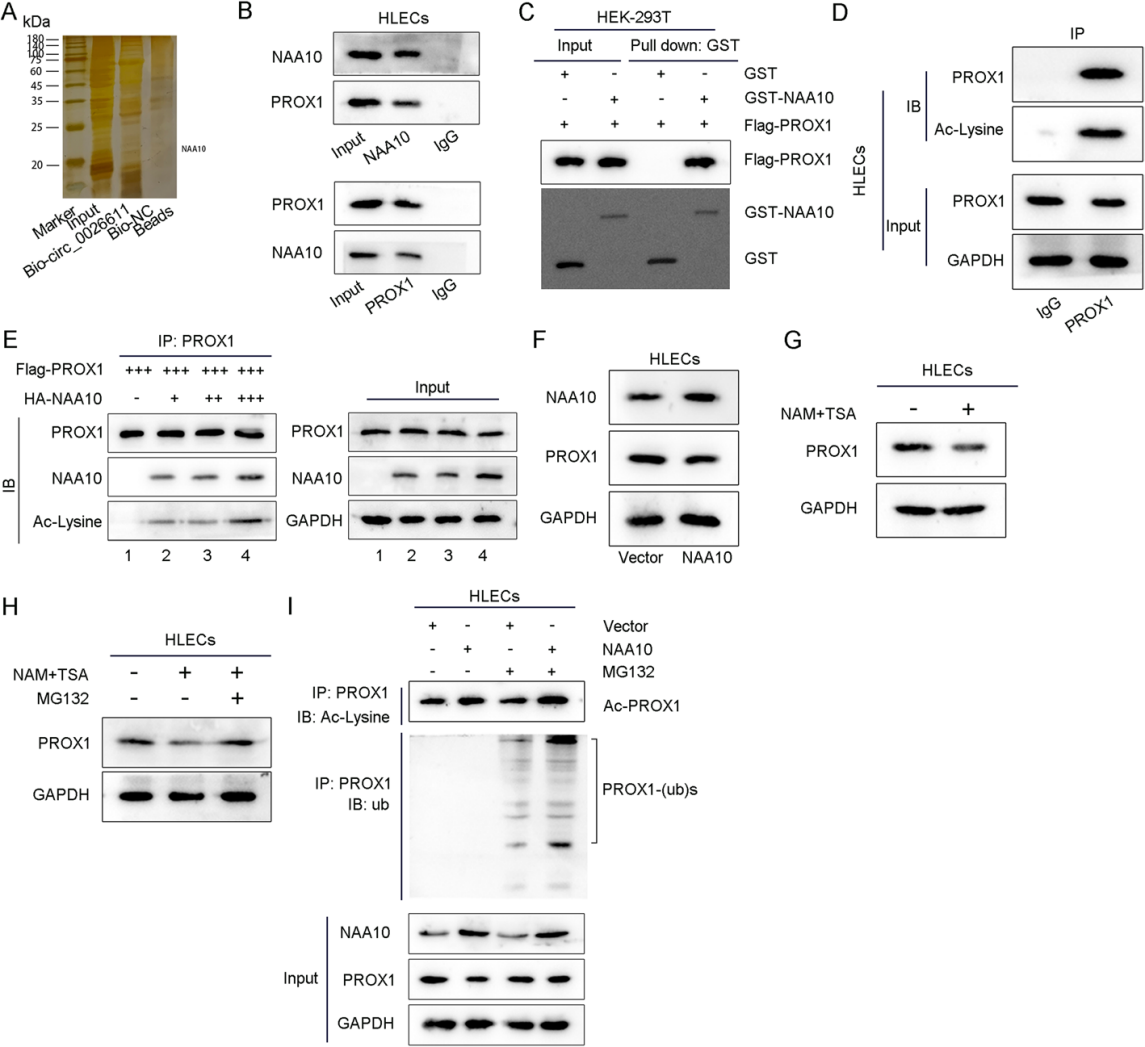
NAA10 interacting with circ_0026611 mediated PROX1 acetylation and promoted its ubiquitination and subsequent degradation. **A** The proteins that could interact with circ_0026611 were explored through RNA pulldown as well as mass spectrometry. **B** CoIP assay verified the interaction between NAA10 and PROX1. **C** The binding between GST-NAA10 and Flag-PROX1 was detected by GST-pulldown assay. **D** CoIP was used to detect whether endogenous PROX1 protein could be acetylated. **E** The effect of NAA10 on PROX1 acetylation was assessed by CoIP assay. **F** Western blot was used to determine the effect of NAA10 on PROX1 protein. **G** Western blot was used to assess the effect of NAM + TSA on PROX1 protein. **H** Protein levels of PROX1 in different treatments. **I** CoIP assay was used to verify the effect of NAA10 on PROX1 protein acetylation and ubiquitination

Then we continued to explore the specific mechanism of circ_0026611 regulating PROX1 protein. It is well known that circRNA could affect the expression or function of certain proteins by interacting with proteins [25, 26]. As revealed by mass spectrometry, NAA10, an acetyltransferase that can acetylate proteins at the posttranslational level, was found to have the potential to interact with circ_0026611 in HLECs (Fig. 5A). Therefore, we speculated that circ_0026611 might interact with NAA10 to regulate PROX1 acetylation and thus affect its ubiquitination and degradation. To verify our speculation, we explored the relationship between NAA10 and PROX1. Coimmunoprecipitation (CoIP) assay was performed, revealing that, in the precipitate of NAA10 antibody, NAA10 and PROX1 proteins could be detected, and PROX1 and NAA10 proteins could be detected in the precipitate of PROX1 antibody, indicating that NAA10 could interact with PROX1 in HLECs (Fig. 5B). Then the binding ability between GST-NAA10 and Flag-PROX1 was assessed. It was found that only GST-NAA10 in the IP group could pull down Flag-PROX1, indicating that NAA10 can be directly combined with PROX1 (Fig. 5C). CoIP was used to determine whether endogenous PROX1 protein could be acetylated. In HLECs, PROX1 and acetylated lysine could be detected in the precipitate of PROX1 antibody, suggesting that PROX1 protein could be acetylated (Fig. 5D). Then, by CoIP assay, we found that NAA10 could interact with PROX1 and thus accelerate PROX1 protein acetylation (Fig. 5E). Next, we continued to explore whether NAA10-induced PROX1 acetylation could affect its ubiquitination and degradation. When NAA10 was overexpressed, the level of PROX1 protein was significantly decreased (Fig. 5F). After nicotinamide (NAM) + Trichostatin A (TSA) treatment stabilized PROX1 acetylation, the protein level of PROX1 was significantly downregulated, indicating that PROX1 acetylation inhibited its expression (Fig. 5G). The protein levels of PROX1 in different treatments were assessed, and we found that PROX1 protein was downregulated under NAM + TSA treatment. The result proved that the PROX1 protein was degraded by the proteasome system after its ubiquitination was promoted by acetylation (Fig. 5H). Finally, CoIP assay was carried out, showing that, after NAA10 overexpression, the level of lysine acetylation in the PROX1 protein pulled down by the PROX1 antibody increased; meanwhile, after inhibiting protein degradation with MG132, the level of PROX1 protein ubiquitination seemed to be increased in the NAA10-overexpressed group, which meant that NAA10 promoted PROX1 ubiquitination and acetylation (Fig. 5I). To sum up, the NAA10 interacting with circ_0026611 could induce the acetylation of PROX1 and promote its ubiquitination and degradation (Additional file 3).

### ESCC exosome circ_0026611 interacted with NAA10 to inhibit the acetylation and ubiquitination of PROX1 in HLECs

**Fig. 6:**
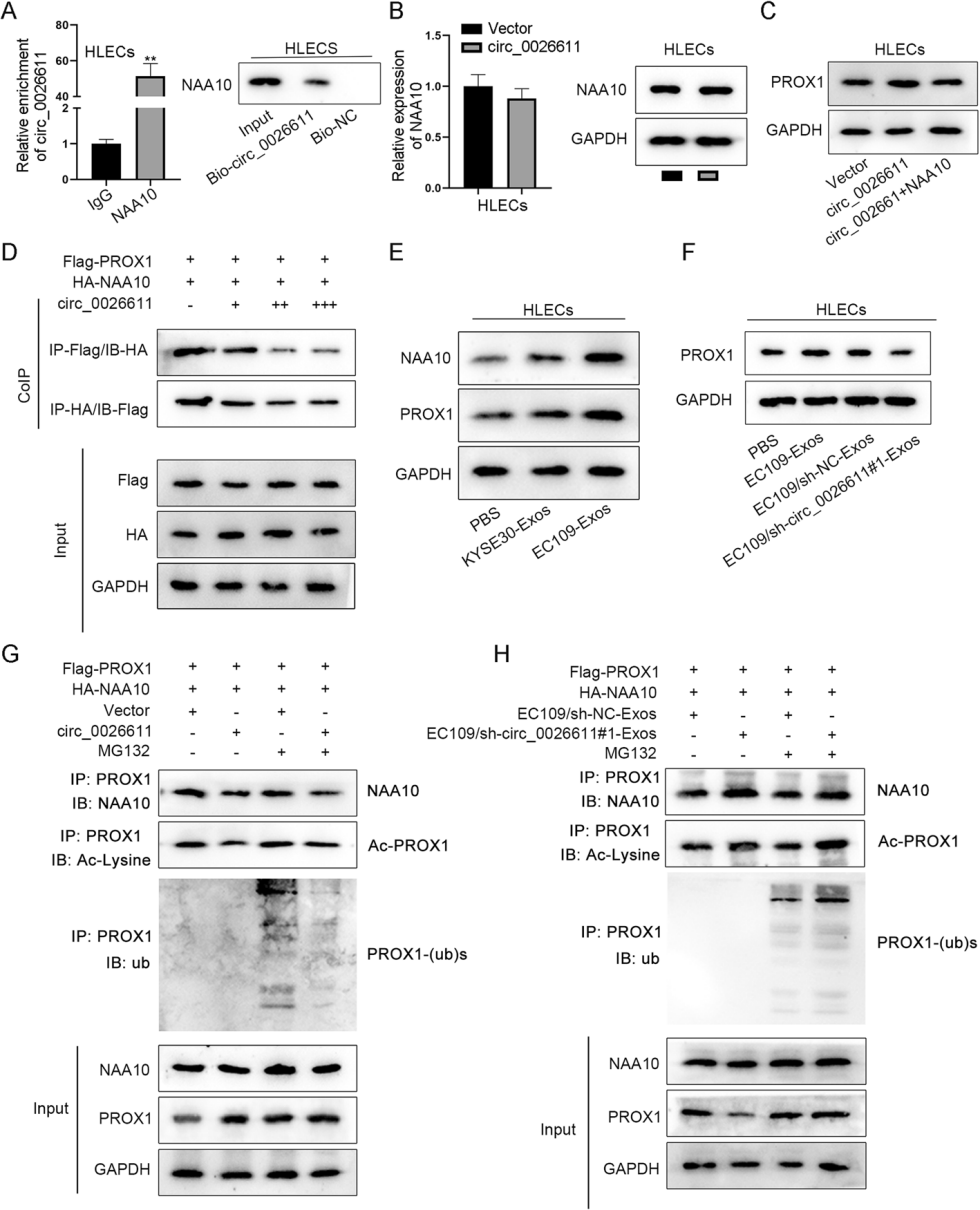
ESCC exosome circ_0026611 interacted with NAA10 to inhibit the acetylation and ubiquitination of PROX1 in HLECs. **A** NAA10 enrichment as well as its protein expression in HLECs. **B** NAA10 expression as well as protein expression affected by circ_0026611. **C** Protein expression of PROX1 in different conditions. **D** The effect of circ_0026611 on the interaction between NAA10 and PROX1 was assessed by CoIP. **E** The expression of NAA10 and PROX1 protein in HLECs under different treatments. **F** Western blot assay was conducted to test the effect of different EC109-Exos treatments on PROX1 protein. **G** CoIP assay was used to determine the effect of circ_0026611 on PROX1 protein acetylation and ubiquitination. **H** CoIP was carried out to assess the effect of EC109-Exos coated with different circ_0026611 expression levels on PROX1 protein. **P < 0.01

Finally, we wanted to know whether circ_0026611 transmitted by ESCC exosomes affected PROX1 protein by interacting with NAA10. We found binding potential between circ_0026611 and NAA10 in HLECs, as the NAA10 antibody could be pulled down by circ_0026611 (Fig. 6A). However, circ_0026611 overexpression barely affected NAA10 expression (Fig. 6B). These findings indicated that circ_0026611 could only interact with NAA10 without influencing its expression. The next step was to explore whether the interaction could influence PROX1 expression. The upregulated PROX1 level caused by circ_0026611 overexpression was reversed by NAA10 overexpression (Fig. 6C). The inhibitory effect of circ_0026611 on the interaction between NAA10 and PROX1 was detected by CoIP (Fig. 6D). The effect of ESCC-derived exosomes on the expression of NAA10 and PROX1 protein in HLECs and the effect of different EC109-Exos treatments on PROX1 protein were analyzed by western blot. CoIP assay was used to assess the effect of circ_0026611 on PROX1 protein acetylation and ubiquitination as well as the effect of EC109-Exos coated with different circ_0026611 expression levels on PROX1 protein. From the results in Fig. 6E–H, we concluded that circ_0026611 delivered by ESCC-derived exosomes inhibited NAA10-mediated PROX1 protein acetylation and ubiquitination, thereby upregulating PROX1 protein expression.

## Discussion

circ_0026611 is a novel circRNA that has not been widely reported in cancer development. circRNA has been demonstrated to exert different regulatory functions in the development of cancers via various mechanisms. For example, circ-CAMK2A can target the pathway constituted by miR-615-5p and fibronectin 1 to exert a promoting impact on lung adenocarcinoma metastasis [27]. circ_0086296, together with miR-576-3p, IFIT1, and STAT1, constitutes a feedback loop that participates in the regulation of atherosclerosis progression, and high circ_0086296 expression was confirmed in the exosomes of serum of patients with atherosclerosis [28]. What we discovered about circ_0026611 was consistent with the previous literature [16], in which its high expression pattern was verified in ESCC cells and the exosomes secreted from the cells. To the best of our knowledge, we are the first to have revealed circ_0026611 as a potential regulatory molecule in ESCC, which may help to enrich the knowledge on this circRNA in regulating cancer malignancy as well as to provide strategic options for ESCC treatment.

LNM has been identified as an independent prognostic factor of ESCC [5, 29]. It is a complicated process comprising multiple steps, including lymphangiogenesis [7]. LNM has been reported to be associated with lymphatic metastasis in cancer [9, 10], including in ESCC [11]. Exosomes, membrane-derived vesicles originating from endosomal multivesicular bodies, have a size range of 20–150 nm, and these vesicles contain protein, lipids, and coding or noncoding RNAs derived from their donor cell cytoplasm and can be taken up by other cells [30]. Studies have found that circular RNA is enriched and stable in exosomes, making it a promising biomarker for cancer diagnosis [19, 31]. On the basis of what we have uncovered, we conclude that ESCC cell-derived exosomes could promote lymphangiogenesis, as well as proliferation, migration, and lymphangiogenesis of HLECs by transferring circ_0026611. On the basis of the findings, we discovered that circ_0026611 could regulate the mRNA and protein levels of VEGFR3 as well as PROX1. Studies have disclosed the important roles of VEGFR3 and PROX1 in the process of lymphangiogenesis [21, 22]. More interestingly, it was found through previous research that PROX1 mediates the regulation of VEGFR3 [23, 24]. Consistent with these findings, we found that circ_0026611 could regulate VEGFR3 in an indirect manner by positively enhancing PROX1 protein in HLECs. Emerging studies have demonstrated that circRNAs can affect the behavior of proteins via direct interactions with them [25, 26]. In this study, NAA10 was discovered to be the protein that may interact with circ_0026611. NAA10 is an acetyltransferase that acetylates proteins at the posttranslational level, and it has been demonstrated by many studies to be a potential prognostic marker for cancer progression [32, 33].

Interestingly, we found that, recently, many reports have demonstrated the relationship between protein acetylation and ubiquitinylation [34–36]. Therefore, we carried out a series of mechanism experiments and found that NAA10 could induce PROX1 acetylation so as to promote its ubiquitination and degradation, and the effect of exosomal circ_0026611/NAA10/PROX1 axis in HLECs was finally verified. We demonstrated that exosomal circ_0026611 derived from ESCC cells could inhibit NAA10-mediated PROX1 protein acetylation and ubiquitination so as to enhance the expression level of PROX1 protein.

## Conclusions

This study suggests that exosomal circ_0026611 contributes to lymphangiogenesis in ESCC by reducing PROX1 acetylation and ubiquitination. As we have illustrated before, as circ_0026611 is a novel circRNA, there has not been sufficient literature to fully support its implications in cancer, let alone ESCC. Therefore, the detailed regulation of this new circRNA as well as its clinical value in the interaction with exosomes and cancer development requires further research, which is our working direction in the near future. It is our hope that this study may provide a novel insight into as well as meaningful biomarkers for the treatment and prognosis of ESCC.

## Supplementary Information

**Additional file 1: Figure S1.** Circ_0026611 indirectly up-regulated VEGFR3 by regulating PROX1 protein after translation. A. Luciferase reporter assay was used to detect the effect of circ_0026611 on VEGFR3 promoter. B. The expression changes of PROX1 and VEGFR3 in HLECs after sh-PROX1#1/2 transfection. C. Luciferase reporter assay was utilized to detect the influence of PROX1 to VEGFR3 transcription. D. Luciferase reporter assay was used to detect the luciferase activity change of VEGFR3 promoter. E. VEGFR3 expression in different conditions was measured. F. Western blot was utilized to detect the CHX treated PROX1 protein change after up-regulating circ_0026611. G. PROX1 protein under MG132 treatment was measured after circ_0026611 up-regulation. **P < 0.01.

**Additional file 2: Table S1.** Related information on sequences.

**Additional file 3. **Original images of western blot and the original image of mass spectrometry in Figure 5A.

## Data Availability

Not applicable.

## Abbreviations

circRNAs: Circular RNAs
cDNA: Complementary DNA
DMEM: Dulbecco’s modified Eagle medium
ECM: Endothelial cell medium
ECL: Enhanced chemiluminescence
ESCC: Esophageal squamous carcinoma
FBS: Fetal bovine serum
GAPDH: Glyceraldehyde-3-phosphate dehydrogenase
HLECs: Human lymphatic endothelial cells
HUVECs: Human umbilical endothelial cells
LNM: Lymph node metastasis
LNs: Lymph nodes
LYVE-1: Lymphatic vessel endothelial hyaluronan receptor 1
mRNAs: Messenger RNAs
MV: Microvesicle
NAA10: *N*-α-acetyltransferase 10
PMX: Particle Metrix
PROX1: Prospero homeobox 1
RT-qPCR: Quantitative reverse transcription real-time polymerase chain reaction
RIP: RNA-binding protein immunoprecipitation
shRNAs: Short hairpin RNAs
TEM: Transmission electron microscopy
VEGF-C: Vascular endothelial growth factor C
VEGF-D: Vascular endothelial growth factor D
VEGFR3: Vascular endothelial growth factor receptor 3
