# Supplementary figures and images for: Exosomal circ_0026611 contributes to lymphangiogenesis by reducing PROX1 acetylation and ubiquitination in human lymphatic endothelial cells (HLECs)
Source: Cell Mol Biol Lett. 2023 Feb 17;28:13. doi: 10.1186/s11658-022-00410-z (PMC9936748; doi:10.1186/s11658-022-00410-z)

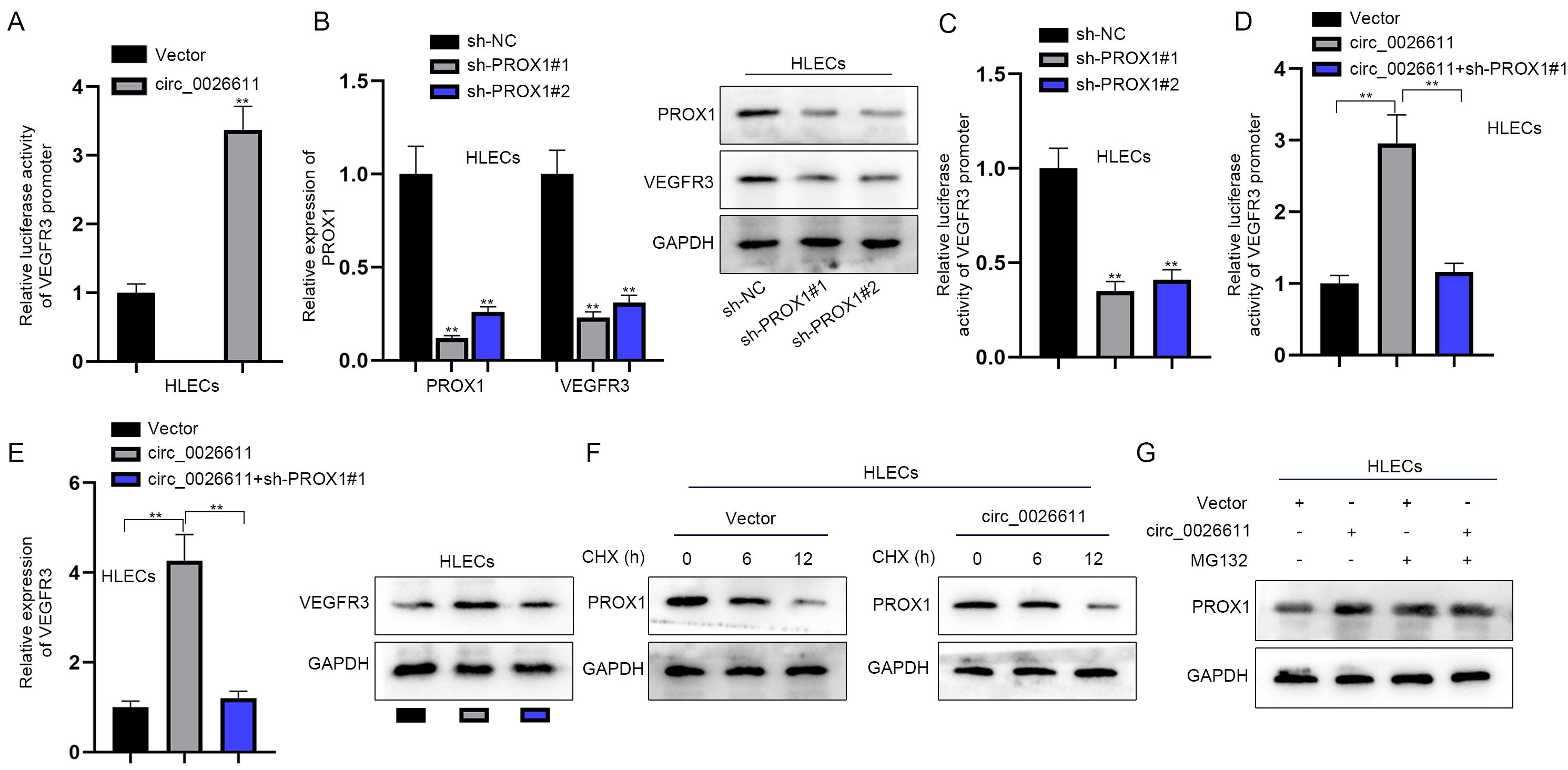

Supplement: Supplementary file 1 — Additional file 1: Figure S1. Circ_0026611 indirectly up-regulated VEGFR3 by regulating PROX1 protein after translation. A. Luciferase reporter assay was used to detect the effect of circ_0026611 on VEGFR3 promoter. B. The expression changes of PROX1 and VEGFR3 in HLECs after sh-PROX1#1/2 transfection. C. Luciferase reporter assay was utilized to detect the influence of PROX1 to VEGFR3 transcription. D. Luciferase reporter assay was used to detect the luciferase activity change of VEGFR3 promoter. E. VEGFR3 expression in different conditions was measured. F. Western blot was utilized to detect the CHX treated PROX1 protein change after up-regulating circ_0026611. G. PROX1 protein under MG132 treatment was measured after circ_0026611 up-regulation. **P < 0.01. [file 11658_2022_410_MOESM1_ESM.tif]

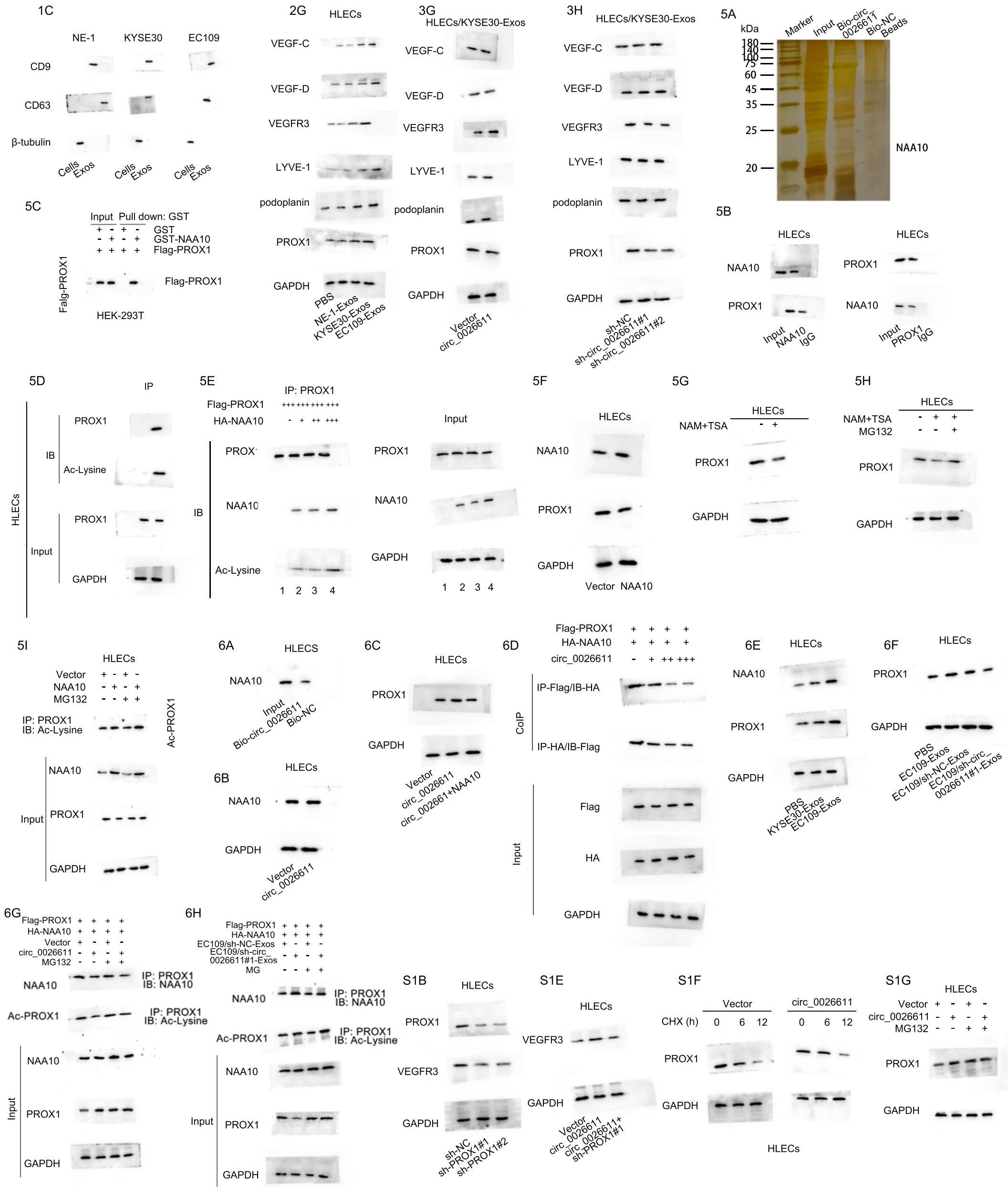

Supplement: Supplementary file 3 — Additional file 3. Original images of western blot and the original image of mass spectrometry in Figure 5A. [file 11658_2022_410_MOESM3_ESM.pdf]
